# A Phase 1, Dose-Ranging Study to Assess Safety and Psychoactive Effects of a Vaporized 5-Methoxy-N, N-Dimethyltryptamine Formulation (GH001) in Healthy Volunteers

**DOI:** 10.3389/fphar.2021.760671

**Published:** 2021-11-25

**Authors:** Johannes Reckweg, Natasha L. Mason, Cees van Leeuwen, Stefan W. Toennes, Theis H. Terwey, Johannes G. Ramaekers

**Affiliations:** ^1^ Faculty of Psychology and Neuroscience, Maastricht University, Maastricht, Netherlands; ^2^ Institute of Legal Medicine, University of Frankfurt, Frankfurt, Germany; ^3^ GH Research, Dublin, Ireland

**Keywords:** 5-MeO-DMT, psychedelic agents, psychoactive, cognition, dose finding

## Abstract

5-Methoxy-N,N-Dimethyltryptamine (5-MeO-DMT) is a tryptamine with ultra-rapid onset and short duration of psychedelic effects. Prospective studies for other tryptamines have suggested beneficial effects on mental health outcomes. In preparation for a study in patients with depression, the present study GH001-HV-101 aimed to assess the impact of four different dose levels of a novel vaporized 5-MeO-DMT formulation (GH001) administered via inhalation as single doses of 2 (*N* = 4), 6 (*N* = 6), 12 (*N* = 4) and 18 mg (*N* = 4), and in an individualized dose escalation regimen (*N* = 4) on the safety, tolerability, and the dose-related psychoactive effects in healthy volunteers (*N* = 22). The psychedelic experience was assessed with a novel Peak Experience Scale (PES), the Mystical Experience Questionnaire (MEQ), the Ego Dissolution Inventory (EDI), the Challenging Experience Questionnaire (CEQ), and the 5-Dimensional Altered States of Consciousness Questionnaire (5D-ASC). Further aims were to assess the impact of 5-MeO-DMT on cognitive functioning, mood, and well-being. Higher doses of 5-MeO-DMT produced significant increments in the intensity of the psychedelic experience ratings as compared to the lowest 2 mg dose on all questionnaires, except the CEQ. Prominent effects were observed following single doses of 6, 12, and 18 mg on PES and MEQ ratings, while maximal effects on PES, MEQ, EDI, and 5D-ASC ratings were observed following individualized dose escalation of 5-MeO-DMT. Measures of cognition, mood, and well-being were not affected by 5-MeO-DMT. Vital signs at 1 and 3 h after administration were not affected and adverse events were generally mild and resolved spontaneously. Individualized dose escalation of 5-MeO-DMT may be preferable over single dose administration for clinical applications that aim to maximize the experience to elicit a strong therapeutic response.

## Introduction

Psychedelic drugs such as mescaline, psilocybin, and lysergic acid diethylamide (LSD) were extensively used in psychiatry before they were placed in Schedule I of the United Nations Convention on Psychotropic Substances in 1971. After decades of suspension, there is now a major revival of human psychedelic research driven by modern brain imaging studies and clinical trials providing safety and efficacy data for the use of psychedelics in the treatment of mental disorders (Carhart-Harris and Goodwin, 2017; Reiche et al., 2018; Carhart-Harris et al., 2021).

5-Methoxy-N,N-Dimethyltryptamine (5-MeO-DMT) is a naturally-occurring tryptamine with high potency, ultra-rapid onset, and short duration of psychedelic effects (Shulgin and Shulgin, 1997). 5-MeO-DMT is predominantly found with other psychoactive molecules in the toxic secretion of the *Bufo alvarius* toad, also called the Sonoran Desert toad or Colorado River toad, but also in certain plants, such as *Dictyoloma incanescens* (Pachter et al., 1959). 5-MeO-DMT was first synthesized in 1936 (Hoshino and Shimodaira, 1936) and while it has a similar molecular structure as the classic psychedelics N,N-Dimethyltryptamine (DMT) and psilocybin, its pharmacological activity might be distinct. 5-MeO-DMT works mainly as a selective serotonin receptor agonist at the 5-HT_1A_ and 5-HT_2A_ receptors, whereby functional dominance of the 5-HT1A subtype has been suggested (Spencer et al., 1987; Winter et al., 2000; Krebs-Thomson et al., 2006; Shen et al., 2010), while DMT and psilocybin (i.e. psilocin) are overall less selective (Ray, 2010) with some data indicating a functional dominance of the 5-HT2A subtype (Vollenweider et al., 1998; Barker, 2018; Madsen et al., 2019).

5-MeO-DMT from natural or synthetic sources has a history of naturalistic use in spiritual or self-exploratory contexts (Weil and Davis, 1994; Davis et al., 2018), and its particular potency and ability to induce so called peak experiences (PE) or mystical experiences has been highlighted (Ott, 1999; Barsuglia et al., 2018). 5-MeO-DMT-related PEs have been described to encompass the experience of “ego dissolution” and an overwhelming sense of “oneness” or “unity” that can elicit an emotional change in perspective (Davis et al., 2018; Uthaug et al., 2019). The occurrence of PEs has been shown to be correlated with therapeutic effects in many of the prior studies with other psychedelic agents, including tryptamines (Garcia-Romeu et al., 2014; Johnson et al., 2014; Bogenschutz et al., 2015; Griffiths et al., 2016; Ross et al., 2016; Roseman et al., 2018). Observational studies on the naturalistic use of 5-MeO-DMT and toad venom containing 5-MeO-DMT have reported that the intensity of the experience is associated with improvements in subjective measures of satisfaction with life and reduction of psychological distress in participants without an underlying mental health condition (Uthaug et al., 2019; Uthaug et al., 2020) and an anonymous web-based survey has reported that the intensity of mystical experiences and higher ratings of spiritual significance and personal meaning is associated with self-reported improvements of self-reported depression and anxiety (Davis et al., 2019). However, it is currently still unknown whether 5-MeO-DMT actually has therapeutic effects in mental disorders, and which dose range of 5-MeO-DMT is needed to potentially elicit a therapeutic response.

This is the first formal prospective clinical study to investigate the safety profile of 5-MeO-DMT and its dose-related effects on states of consciousness. Most available information on 5-MeO-DMT is derived from retrospective surveys or internet fora such as Erowid (erowid.org) or observational studies (Uthaug et al., 2019; Uthaug et al., 2020), but since those reports describe the use of material of largely unknown quantity, quality, and purity, which is often naturally-sourced, and with likely highly variable administration methods and bioavailability, no firm conclusions can be drawn from those studies, including on dose-response effects on states of consciousness and dose-related safety aspects. What has been suggested, however, is that 5-MeO-DMT is non-addictive, and with a low potential for abuse (Davis et al., 2018). Also, the onset of psychoactive effects after inhalation was shown to occur within 1–50 s (Uthaug et al., 2019), with an overall duration of effects of up to about 30 min (Davis et al., 2018). In contrast to other psychedelic substances, there seems to be very little build-up of tolerance to the effects of 5-MeO-DMT (Schlemmer et al., 1977; Strassman and Qualls, 1994; Strassman et al., 1996).

The current study (GH001-HV-101) primarily aimed to investigate safety and tolerability and the psychoactive effects of a novel vaporized 5-MeO-DMT formulation (GH001) in healthy volunteers in a controlled clinical setting. Further aims were to assess the impact of 5-MeO-DMT on cognitive functioning, mood, and well-being. The study evaluated single, ascending doses of 2, 6, 12, and 18 mg 5-MeO-DMT administered via inhalation as well as individualized dose escalation (IDE) of 5-MeO-DMT at doses ranging from 6 to 18 mg. The overall aim of the study was to determine the dosing regimen of 5-MeO-DMT that would achieve a peak experience in healthy volunteers. That aim is relevant for future dose selection in clinical populations, because strong associations between psychedelic peak experiences and therapeutic effects have been reported for other tryptamines (Garcia-Romeu et al., 2014; Johnson et al., 2014; Bogenschutz et al., 2015; Griffiths et al., 2016; Ross et al., 2016; Roseman et al., 2018). Higher doses were contemplated to occasion stronger psychoactive effects, with the IDE contemplated to elicit the strongest effects and having the highest potential to cause a peak experience. Due to the short-lived nature of psychoactive effects, no clinically-relevant change in scores on cognitive measures were expected.

## Materials and Methods

### Participants

A total of 22 healthy volunteers (9 female, 13 male) aged 18–42 years (*Mean* = 29, *SD* = 6.08) were recruited through advertisements posted at Maastricht University and on social media. Each participant needed to have at least two previous psychedelic experiences with substances from the classes of tryptamines, ergolines, or phenethylamines, but not within the previous 4 weeks. A summary of previous drug use among participants can be found in Supplementary Table S1. Exclusion criteria are provided in the Supplementary Information. Participants gave written informed consent and received standard monetary compensation for their participation in the study. The study was approved by the Dutch Central Committee on Research Involving Human Subjects (CCMO) and the Medical Ethics Committee of the Academic Hospital of Maastricht and Maastricht University and conducted according to the principles of Good Clinical Practice (GCP) and the code of ethics on human experimentation established by the declaration of Helsinki 1964) and amended in Fortaleza (2013). The study was registered in the Dutch CCMO-register (NL.67778.068.18), EudraCT (2018-003632-68), and clinicaltrials.gov (NCT04640831).

### Design

The phase 1 study was comprised of two single-arm parts, where Part A was a single-ascending dose study and Part B used an individualized dose escalation regimen (IDE). The dose levels of 5-MeO-DMT (as GH001) investigated in Part A were 2, 6, 12, and 18 mg given to groups of four different volunteers (*N* = 4) at each dose level. In the 2 mg dose condition only threshold effects were expected. Before allowing dosing at a higher dose level, a Study Safety Group (SSG) performed an evaluation of all available safety data, data on cognitive tests, and data available on whether the participants achieved a PE. After completion of the planned four participants of the 6 mg dose group, the SSG recommended to add two more participants in this dose group because of motion-based artefacts in vital sign monitoring, performed with the Caretaker device (6 mg: total *N* = 6). Doses in between the pre-defined dose levels (i.e., 4, 9, and 15 mg) were available for recommendation, if required, but were not used. Each dose group had to contain at least one male and one female participant. In Part B, an IDE regimen of 6, 12, and 18 mg was applied, where at least one and up to three doses of 5-MeO-DMT (as GH001) interspaced at 3 h were given on a single day. The decision to move on to the next dose was guided by an evaluation of whether the participant achieved a PE at the previously administered dose and whether the previously administered dose was safe. If no PE had been achieved, the next dose was administered, up to the maximum of three doses, after consultation with and consent from the participant. Participants in part A and B were not informed on actual identity of the study drug and the actual dose levels that were administered during participation, but were aware that all doses were active (part A and B). Participants were informed that they received a tryptamine psychedelic and were extensively informed on the subjective effects of treatment, the duration of the effects and potential adverse events. This procedure was followed to prohibit participant bias on the drug experience based on searches on the internet and to keep potential expectancy effects across participants in part A and B similar. After completion of the study, participants were informed about the identity of the study drug and the dose level(s) to which they had been exposed.

For both parts, the study was comprised of four visits. On the first visit (Visit A), the medical screenings as well as baseline measurements were performed. The second visit (Visit B) was the administration day on which the participants inhaled the study drug. For the third visit (Visit C), the participants were contacted by phone the day after the administration. For the fourth visit (Visit D), the participants came back to the clinic 7 days after the administration. To avoid expectancy effects, only after completion of the study, participants were informed about the identity of the study drug and the dose level(s) to which they had been exposed.

### Study Drug Administration

GH001 (GH Research, Dublin, Ireland) is an investigational drug product based on a proprietary formulation of highly pure, GMP pharmaceutical grade 5-MeO-DMT for administration via inhalation. Liquid GH001 was administered after a standardized vaporization procedure using the Volcano Medic Vaporization System (Storz and Bickel, Germany), approved in Europe, Australia, and Canada for medical use with cannabinoids (Gieringer et al., 2004; Hazekamp et al., 2006; Abrams et al., 2007). The device consists of a hot air generator, which allows formation of an aerosol from GH001, and a detachable valve balloon (3 L) from which the aerosol is inhaled by the participant with a single breath. Participants were instructed and guided to hold their breath for 10 s before exhaling.

### Adverse Events

Adverse events (AEs) were recorded from inclusion into the study until the end of the study on day 7. AEs were coded according to MedDRA version 22.

### Subjective Ratings of Psychedelic Effects

Subjective psychedelic effects were retrospectively rated at 2 h after the administration representing the participant’s experience during acute 5-MeO-DMT exposure. Subjective ratings included the Peak Experience Scale (PES), duration of experience, the Ego Dissolution Inventory (EDI) (Nour et al., 2016), the Mystical Experiences Questionnaire (MEQ) (Studerus et al., 2010; Barrett et al., 2015), the Challenging Experiences Questionnaire (CEQ) (Barrett et al., 2016) and the 5-Dimensional Altered States of Consciousness Questionnaire (5D-ASC) (Studerus et al., 2010). Detailed descriptions of the rating scales and their dependent variables are provided in the Supplementary Information.

### Subjective Measures of Mood and Well-Being

Subjective measures of mood and well-being included the Depression, Anxiety and Stress Scale (DASS-21) (Henry and Crawford, 2005), the Satisfaction with Life Scale (SWL) (Diener et al., 1985), the Five Facets Mindfulness Questionnaire (FFMQ) (Baer et al., 2006), the Clinician-Administered Dissociative State Scale (CADSS) (Bremner et al., 1998) and the Brief Psychiatric Rating Scale (BPRS) (Overall and Gorham, 1962). Full description of these measures is provided in the Supplementary Information.

### Cognitive Tests

A battery of cognitive tests included the Psychomotor Vigilance Task (PVT) (Lim and Dinges, 2008), the Prospective Memory Task (PMT) (Ramaekers et al., 2009) and the Digit Symbol Substitution Task (DSST) (Royer and Janowitch, 1973). Full description of these tests and their dependent variables are given in the Supplementary Information.

### Vital Signs and Blood Samples

The Caretaker 4 (Caretaker Medical EMEA, United Kingdom) was used to monitor vital signs of the participant throughout the test day. The device allowed for remote monitoring of vital signs including a continuous non-invasive beat-by-beat blood pressure, heart rate, oxygen saturation, temperature, respiration rate, body temperature, and an electrocardiogram (ECG). The medical supervisor continuously monitored the data generated by the Caretaker 4 through integration via a secure Android App. The monitoring system however was not designed to generate standardized high quality research data as movement artifacts during and after exposure to 5-MeO-DMT easily occurred. Therefore, manual cuff-based measurements (Omron M6) of blood pressure and heart rate were added for the 12, 18 mg, and IDE group. These assessments were done at 30 min before and at 1 and 3 h after dosing with 5-MeO-DMT, while participants were in supine position after 4 min of rest. Standard assessments were not collected during peak exposure to 5-MeO-DMT to avoid interference with the psychedelic experience.

Blood and urine samples for laboratory safety measures were collected at screening, at the end of the test day, and on the final visit. Additionally, for drug concentration analyses, blood samples were taken 1 and 3 hours after administration, to ascertain the elimination of 5-MeO-DMT and its metabolite bufotenine (5-hydroxy-N,N-dimethyltryptamine). 5-MeO-DMT was determined in serum (200 µL) using liquid chromatography-tandem mass spectrometry (LC-MS/MS) after liquid-liquid extraction. The sensitivity of the validated method was high with a lower limit of quantitation (LLOQ) of 0.014 ng/ml and a limit of detection (LOD) of 0.005 ng/ml. In Part B, only the 3-h post administration samples were taken. The present study did not assess the full pharmacokinetic profile of 5-MeO-DMT exposure, to avoid interference of a blood draw procedure with the psychedelic experience.

### Statistical Analysis

ANOVAs with a single factor Dose (5 levels: 2, 6, 12, 18 mg and IDE) were performed on measures of the psychedelic experience. A generalized linear model repeated-measures (GLM) analysis of variance (ANOVA) with *Dose* (5 levels), *Time* (3 levels: Baseline, after administration, and 7-days follow up) and their interaction (*Time × Dose*) was performed on the cognitive tests and the subjective well-being questionnaires. For dose contrasts, the 2 mg group, where only threshold psychoactive effects occurred, was used as the comparison group. For Part B, the individual scores after the final administration were included in the analysis. Vital sign data were subjected to a generalized linear model repeated-measures ANOVA with the factors Dose (5 levels), Time (3 levels: Baseline, 1 and 3 h after administration) and their interaction. The alpha criterion of statistical significance was set at *p* = 0.05. The Greenhouse-Geisser correction was used if the sphericity assumption was violated. All analyses were exploratory of nature and were performed using IBM SPSS Statistics for Windows, Version 26.0.

## Results

### Psychedelic Experience

**FIGURE 1:**
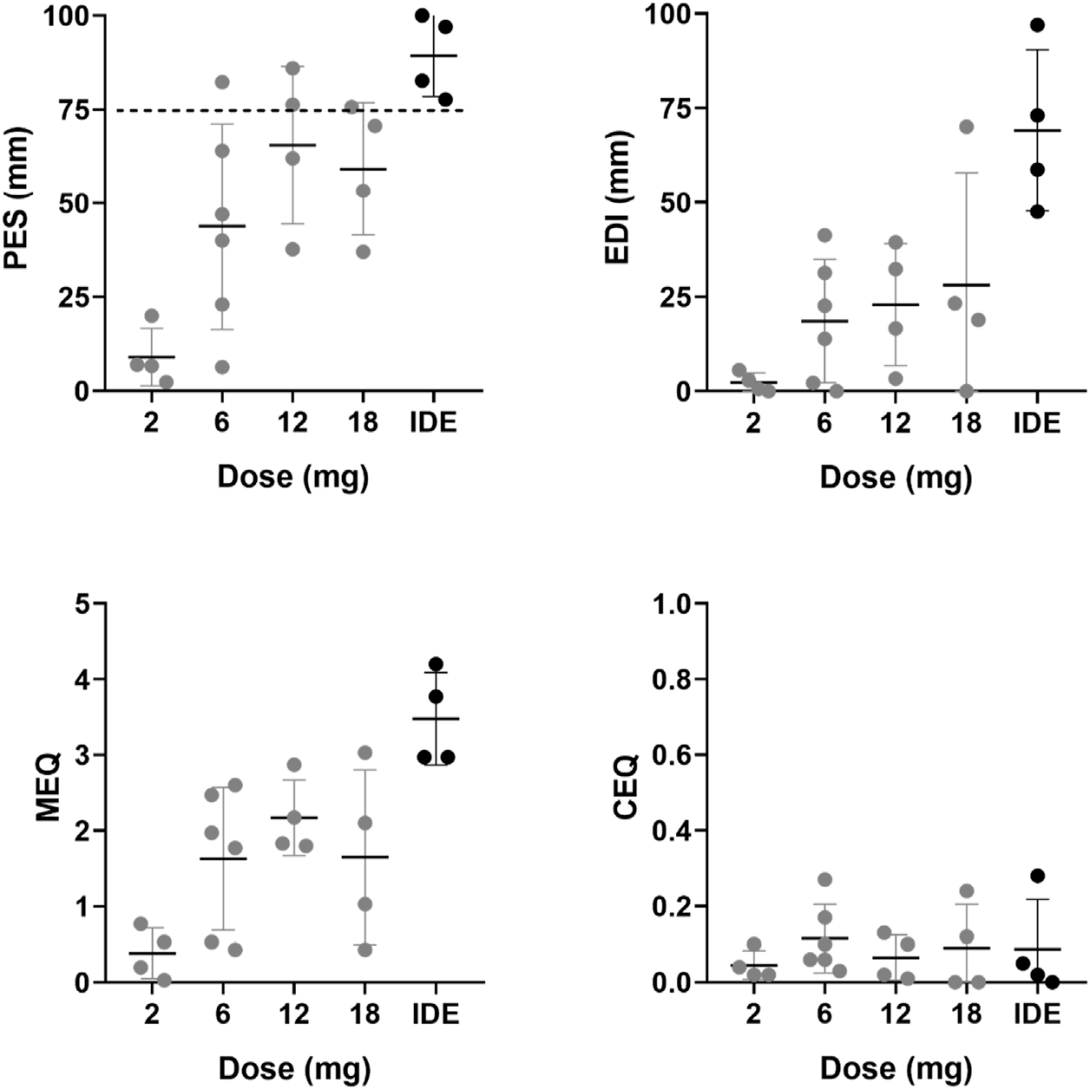
Mean (SE) and individual retrospective ratings of the acute psychedelic experience (PES, EDI, MEQ, CEQ) per dose level.

**FIGURE 2:**
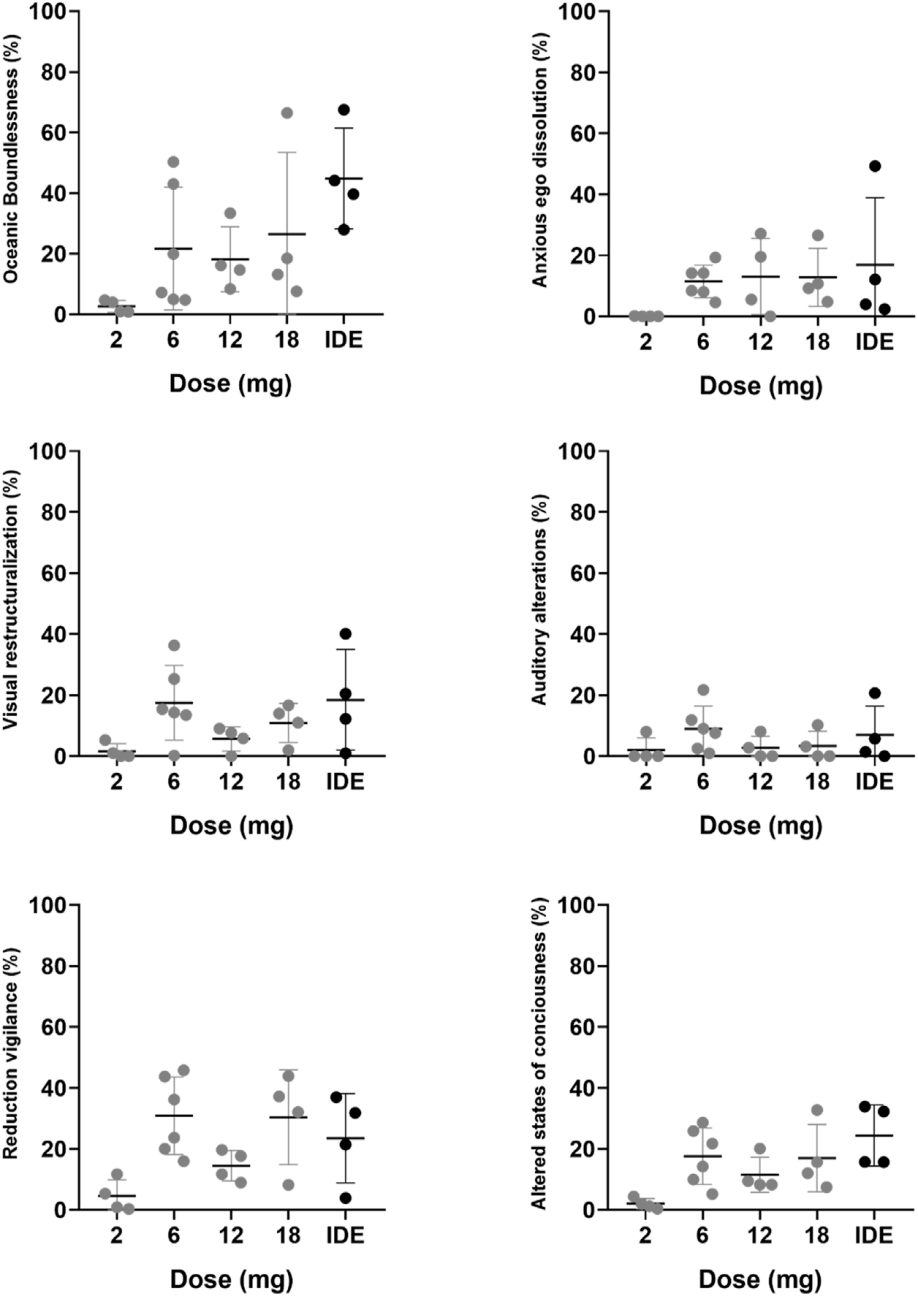
Mean (SE) and individual retrospective ratings of the acute psychedelic experience per dose level for 5 key-dimensions on the 5D-ASC and the overall score.

Mean (SE) and individual rating of the PES, EDI, MEQ, and CEQ are shown in Figure 1. Mean (SE) and individual rating of the key-dimensions of the 5D-ASC are shown in Figure 2. ANOVAs indicated a significant effect of 5-MeO-DMT Dose on ratings of the PES (F_4,17_ = 9.302, *p* < .001, η_p_^2^ = 0.686), EDI (F_4,17_ = 6.925, *p* = .002, η_p_^2^ = 0.62), MEQ (F_4,17_ = 8.026, *p* = .001, η_p_^2^ = 0.654), and Reduction of Vigilance as assessed with the 5D-ASC (F_4,17_ = 4.023, *p* = .018, η_p_^2^ = 0.486). The effects of dose on ratings of Oceanic Boundlessness approached significance (F_4,17_ = 2.901, *p* = .053, η_p_^2^ = 0.406). CEQ ratings were not affected by Dose. Planned contrasts consistently indicated higher mean ratings of the psychedelic experience at higher doses as compared to the lowest GH001 dose of 2 mg. A summary of statistics associated with dose contrasts is given in Supplementary Table S2. In case of PES and MEQ ratings, all doses elicited significantly higher ratings as compared to the lowest dose.

In Part A, one participant achieved a PE (i.e., PES rating ≥75%) in the 6 mg dose group, two participants achieved a PE in the 12 mg dose group, and one participant achieved a PE in the 18 mg dose group. In Part B, applying the IDE, all participants achieved a PE, whereby one participant achieved a PE after the first dose (6 mg), after which no further doses were administered, two participants achieved a PE after the second administration (6 mg + 12 mg), and one participant reached a PE after the third administration (6 mg + 12 mg + 18 mg). EDI ratings significantly increased only after comparing the score at the end of the individualized dose escalation with the 2 mg scores from Part A. 5D-ASC ratings of Oceanic Boundlessness significantly increased at the end of the individualized dose escalation, ratings of Visual Restructuralization significantly increased after the 6 mg dose and at the end of the IDE, and ratings of Reduced Vigilance significantly increased after 6 and 18 mg, and during the IDE. The mean (SD) estimated duration of the psychedelic experience with 5-MeO-DMT was 13.5 min (5.8) according to observations of the investigator and 17.7 min (13.3) according to subjective estimation of the participants.

### Psychiatric Ratings and Cognition

**FIGURE 3:**
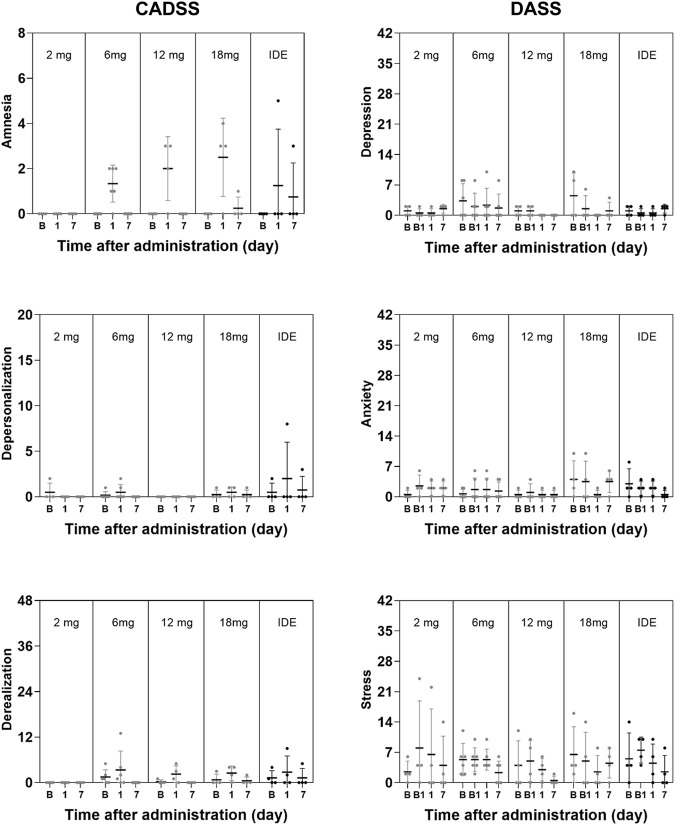
Mean (SE) and individual CADSS and DASS ratings per dose level.

**FIGURE 4:**
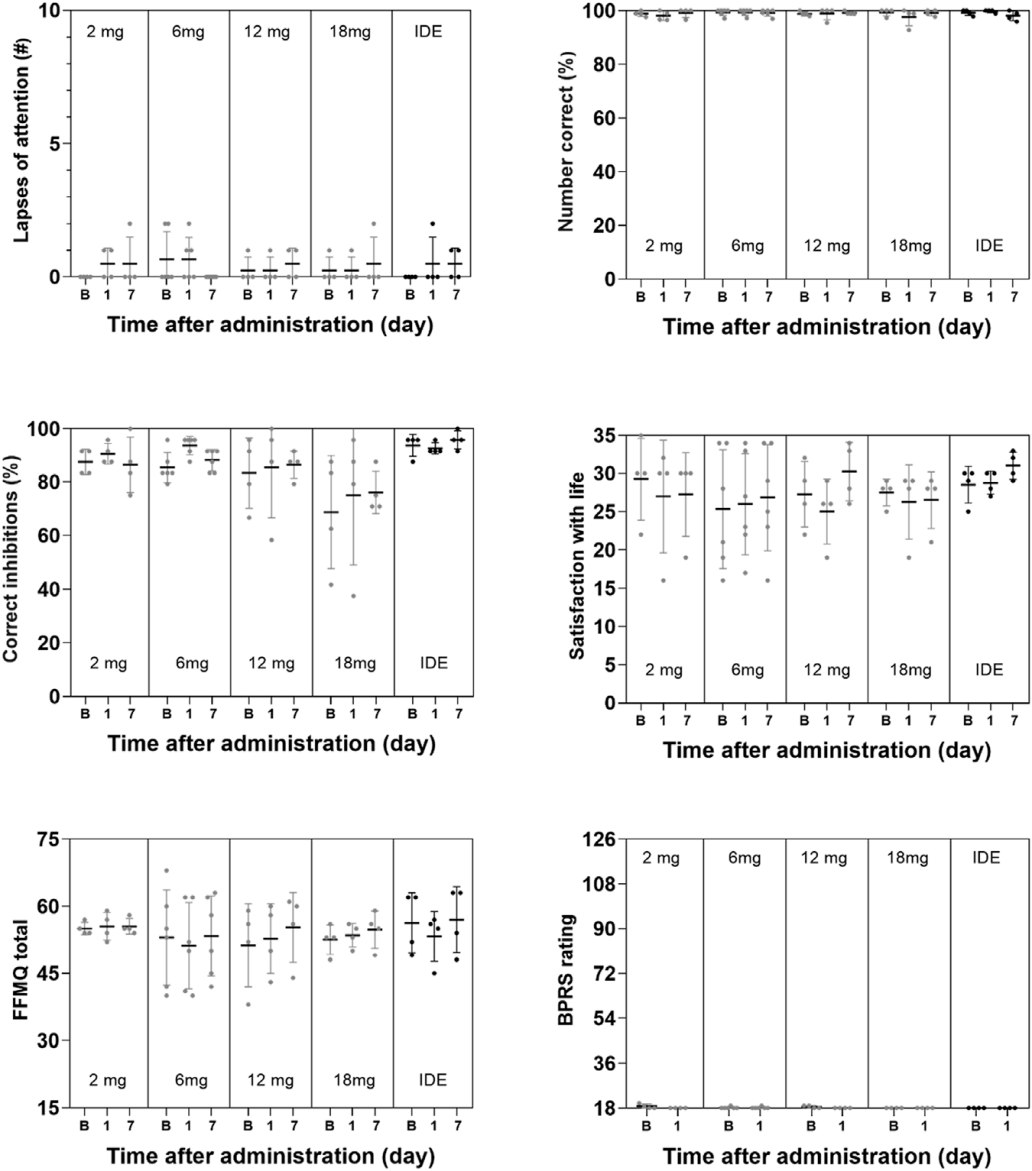
Mean (SE) and individual lapses of attention in the PVT, number of correct responses in the DSST, number of correct inhibitions in the PVT, ratings of satisfaction with life, mindfulness (FFMQ) and BPRS in every dose condition.

GLM analyses did not indicate a significant effect of *Dose* or the interaction of *Time × Dose* on any subjective measure of well-being, mood and cognition. The only exception being a significant interaction effect of *Time × Dose* on the Describe subscale in the FFMQ (F_8,34_ = 4.63, *p* = .001, η_p_^2^ = 0.521) that were considered to be of no clinical relevance given that overall changes on this parameter were minor. The factor *Time* reached significance for the SWLS and the BPRS, and the CADSS subscales of Amnesia and Derealization, as well as overall CADSS rating. A summary of mean (SE) scores on psychiatric and cognitive measures are given in Figures 3, 4.

### Safety and Tolerability

The SSG concluded that the dose range of 2–18 mg was safe and well-tolerated in both single-dose administration in Part A and as part of the individualized dose escalation in Part B. No adverse events (AEs) led to withdrawal, and no Serious AEs (SAEs) were reported. All AEs resolved spontaneously. All drug-related AEs were mild except an AE of “Heart rate increased” at the 12 mg dose level and an AE of “fatigue” in one participant in Part B (after a 12 mg dose), which were moderate in intensity. A summary of AEs with a relationship to the investigational product reported as definite, probable, or possible, or where relationship code is missing is given in Table 1. ANOVA revealed no significant main effects of *Dose, Time* and *Time × Dose* on measures of systolic and diastolic blood pressure. Heart rate was not affected by *Dose* and *Time × Dose* but did reveal a main effect of *Time* (F_2,34_ = 7.18, *p* = .003, ηp2 = 0.297), reflecting a mild decrease in heart rate from baseline to 3 h after administration (non-clinically significant, heart rate within the normal range). A summary of mean (SE) systolic and diastolic blood pressure and heart rate at baseline and at 1 and 3 h after administration is given in Table 2.

**TABLE 1:** Adverse event reported with a relationship to the investigational product as definite, probable, or possible, or where relationship code is missing, by MedDRA System Organ Class and Preferred Term: All Participants by Dose.

Severity	System Organ Class Preferred Term	Part A				Part B	
		2 mg	6 mg	12 mg	18 mg	6 mg	12 mg
Mild	Ear and labyrinth disorders						
	Hyperacusis	—	—	—	1	—	—
	Eye disorders						
	Vision blurred	1	—	—	—	—	—
	Gastrointestinal disorders						
	Nausea	2	1	—	1	1	1
	General disorders and administration site conditions						
	Fatigue	—	—	—	1	—	—
	Feeling hot	—	1	—	—	—	—
	Nervous system disorders						
	Clumsiness	—	1	—	—	—	—
	Head discomfort	—	—	—	—	—	1
	Headache	—	2	—	1	—	1
	Psychiatric disorders						
	Abnormal dreams	—	—	—	1	—	—
	Anxiety	—	1	1	—	—	—
	Confusional state	—	1	—	—	—	—
	Euphoric mood	—	1	—	—	—	—
	Flashback	—	—	—	1	—	—
		Hallucination	—	—	—	1	—	—
		Insomnia	—	—	—	1	—	—
		Mental fatigue	—	—	—	1	—	—
Moderate	General disorders and administration site conditions						
	Fatigue	—	—	—	—	—	1
	Investigations						
	Heart rate increased	—	—	1	—	—	—

**TABLE 2:** Mean (SE) systolic blood pressure (SBP), diastolic blood pressure (DBP and heart rate (HR) in every 5-MeO-DMT dose condition at baseline and 1 and 3 h after administration. IDE = individualized dose escalation condition. Vital signs assessment in the 2 and 6 mg condition were deduced from continuous Caretaker monitoring data. Vital signs in the 12mg, 18 mg and IDE conditions were collected at discrete time points using standard assessments.

Dose	—	SBP (mmHg)			DBP (mmHg)			HR (bpm)		
		Baseline	1 h	3 h	Baseline	1 h	3 h	Baseline	1 h	3 h
2 mg	Mean	110	112	106	71	68	67	74	71	66
—	SE	3.80	1.84	2.92	3.12	1.65	1.08	4.87	6.34	6.26
6 mg	Mean	120	115	126	75	69	76	78	69	71
—	SE	2.92	6.02	3.67	1.54	4.89	2.74	4.74	4.00	2.75
12 mg	Mean	116	110	119	78	70	71	72	72	62
—	SE	4.42	8.81	3.45	3.32	3.48	3.20	7.49	7.36	3.88
18 mg	Mean	118	116	118	73	76	70	71	68	66
—	SE	9.31	10.17	7.20	5.72	5.45	5.74	7.92	6.54	6.10
IDE	Mean	116	125	124	79	77	77	67	68	64
—	SE	4.78	3.68	4.95	2.87	2.80	3.64	3.76	2.87	1.91

### Concentrations of 5-MeO-DMT and Bufotenine

Plasma concentrations of 5-MeO-DMT at 1 and 3 h after administration were very low at 1 h and barely measurable at 3 h (Table 3). Bufotenine concentrations were below the limit of detection (0.006 ng/ml) for all participants at all timepoints.

**TABLE 3:** Mean (range) of 5-MeO-DMT (ng/ml) concentrations at 1 and 3 h after administration.

	Part A 2 mg (*n* = 4)	Part A 6 mg (*n* = 6)	Part A 12 mg (*n* = 4)	Part A 18 mg (*n* = 4)	Part B 6 mg (*n* = 4)	Part B 6 mg, 12 mg (*n* = 3)	Part B 6 mg, 12 mg, 18 mg (*n* = 1)
1 h	0.37 (0.03–0.69)	0.35 (0.09–0.64)	0.20 (0.13–0.29)	0.97 (0.15–2.42)	—	—	—
3 h	0.03 (<LLOQ-0.04)	0.02 (<LLOQ-0.06)	<LLOQ (<LLOQ)	0.08 (<LLOQ-0.24)	0.03 (<LLOQ-0.09)	0.03 (<LLOQ-0.05)	0.05 (0.05–0.05)

## Discussion

The main aim of the current study was to assess the safety and tolerability of a novel vaporized 5-MeO-DMT formulation (GH001) administered via inhalation as single doses and in an individualized dose escalation and to identify the dosing regimen of 5-MeO-DMT that would elicit a peak experience as assessed with a novel PES scale. In addition, the current study aimed to qualify and quantify the altered state of consciousness as a function of the 5-MeO-DMT dose with validated questionnaires such as the EDI, 5D-ASC, MEQ, and CEQ. Measures of cognitive function, mood and well-being were taken at baseline, at 2 h, and at 7 days after dosing to monitor acute and subacute effects of 5-MeO-DMT on the affective and cognitive state. Higher doses of 5-MeO-DMT produced significant increments in the mean intensity of the psychedelic experience as compared to the lowest 2 mg dose on all questionnaires, except the CEQ. Prominent effects were observed following single doses of 6, 12, and 18 mg, while most peak experiences were observed following individualized dose escalation of 5-MeO-DMT doses. Measures of cognition, mood, and well-being were not affected by 5-MeO-DMT. Vital signs at 1 and 3 h after administration were not affected and adverse events were generally mild and spontaneously resolving. It should be noted that Phase 1 studies are exploratory by nature and that current findings need replication in future studies with larger samples sizes.

Overall, 5-MeO-DMT elicited a substantial psychedelic experience as retrospectively assessed with all questionnaires, except the CEQ. All doses elicited significantly higher PES and MEQ ratings as compared to the lowest dose of 2 mg. In total, eight participants reported a peak experience (i.e., PES rating ≥75%): one participant at the 6 mg dose, two participants at the 12 mg dose, one participant at the 18 mg dose, and all four participants in the individualized dose escalation group. EDI and 5D-ASC ratings generally differed from the lowest 2 mg dose after IDE. Our data suggest that the 5-MeO-DMT dose needed to achieve a peak experience largely varies between individuals. Variability was prominent in the single dose conditions but also in the IDE dose condition. During the latter, peak experiences were achieved in all participants but at different individual doses [after a single dose of 6 mg (*N* = 1), after inhalation of 6 and 12 mg (*N* = 2) and after inhalation of 6, 12, and 18 mg (*N* = 1)]. This suggests that individualized dose escalation of 5-MeO-DMT adequately administered via inhalation is an efficient way to tailor the individual doses needed to reach a peak experience. High inter-individual variability of psychoactive effects after 5-MeO-DMT administration was also described in an observational study (Uthaug et al., 2020), were it was noted that the magnitude of the psychedelic experience was not significantly correlated to the total dose of 5-MeO-DMT that participants received (individual doses ranging from 3 to 24 mg, total dose of up to 4 administrations ranging from 17 to 61 mg). Of note, in our study, peak experiences with 5-MeO-DMT were not considered as challenging by participants as evidenced by very low CEQ and Anxious Ego Dissolution ratings. This suggests that 5-MeO-DMT administered as the inhaled GH001 formulation caused little distress.

The current study was the first to employ the PES, which was developed to provide a quick, accurate and easy to use scale for monitoring the magnitude of psychoactive effects after administration of 5-MeO-DMT. The scale focuses on three dimensions of a psychedelic experience, i.e., intensity, loss of control, and profoundness (Barrett et al., 2015; Nour et al., 2016). As such, the scale integrates central aspects of the MEQ (i.e., profoundness, intensity) as well as the EDI and 5D-ASC (i.e., loss of control/ego dissolution and intensity). Despite its brevity, it appeared to be a relevant indicator of the occurrence of peak experiences in eight individuals that were not always recognized in ratings of the more complex MEQ, EDI, and 5D-ASC scales. For example, in the present study, only three individuals achieved a rating >60% across all subscores of the MEQ (i.e., MEQ rating >3), which is the MEQ threshold for a mystical experience (Barrett et al., 2015; Barsuglia et al., 2018). Likewise, only 4 individuals achieved EDI ratings and oceanic boundlessness ratings above 60% of the total score. The MEQ, EDI and 5D-ASC scales capture many aspects of a psychedelic experience that seem not to be typical for the intense, short-lasting psychoactive effects of 5-MeO-DMT when used in the clinical setting, and those scores may be more appropriate in the spiritual or self-exploratory contexts of naturalistic use. For example, among visitors of a psychospiritual retreat program in Mexico (Barsuglia et al., 2018) who received 50 mg of toad venom containing 5-MeO-DMT, over 75% had “a complete mystical experience” (≥60% on all MEQ subscales). The PES however appears to offer a selective and distinct addition to existing questionnaires for use in the clinical setting that can rapidly capture the occurrence of a peak experience. However, further validation of the PES and its performance relative to other scales is required.

As expected, 5-MeO-DMT formulated as GH001 did not elicit any short-term or long-term changes in memory, attention, and cognitive function. Even 2 hours after the administrations, participants did not perform significantly better or worse compared to baseline or 1 week after administration. These results are in line with the notion that psychoactive effects of 5-MeO-DMT are short-lasting and that cognitive and psychomotor functions quickly return to baseline after administration. These findings further attest to the safety profile of pharmaceutical grade 5-MeO-DMT if adequately administered in a controlled setting and suggest the safety of 5-MeO-DMT in relation to day-to-day operations requiring skilled performance. These findings are also in line with the level of 5-MeO-DMT concentrations in blood that were low already 1 h after administration and were barely measurable at 3 h after administration, even after the individualized dose escalation. This suggests that 5-MeO-DMT is rapidly metabolized and eliminated from the body without causing any relevant accumulation after successive doses. A lack of accumulation has previously been reported for DMT by (Strassman et al., 1996) who found no significant residual build-up of DMT over the course of four consecutive intravenous administrations, 30 min apart. This rapid elimination has also been shown for 5-MeO-DMT in mice and rats (Shen et al., 2010). It should be noted however that the present study provides limited information on the progression of 5-MeO-DMT concentration over time and that dedicated studies are needed to establish a full pharmacokinetic profile of 5-MeO-DMT.

Contrary to previous reports from surveys and observational studies (Davis et al., 2019; Uthaug et al., 2019; Uthaug et al., 2020), no improvement in measures of mood and wellbeing after the administration of 5-MeO-DMT was found; however, such improvements would also not have been expected as the present study selected healthy volunteers with minimal BPRS ratings at baseline. Many participants in the survey (Davis et al., 2019) self-reported having been diagnosed with depression (41%) or anxiety (48%) and most self-reported that these conditions were improved following 5-MeO-DMT use. Likewise, many participants in observational studies (Uthaug et al., 2019; Uthaug et al., 2020) reported psychological self-exploration and problem-solving as their main motives for using 5-MeO-DMT. The absence of change in mental health outcomes of participants in the present study therefore does not contradict with previous reports.

The present study provides relevant information on the dose range and regimen of the inhaled GH001 formulation of 5-MeO-DMT that can be expected to elicit a psychedelic peak experience. Previous research with other psychoactive tryptamines has repeatedly demonstrated that the magnitude of a psychedelic experience is a strong predictor of a positive therapeutic response in patients suffering from depression (Roseman et al., 2018; Palhano-Fontes et al., 2019; Romeo et al., 2021). This may be the case for 5-MeO-DMT as well, although a confirmation needs to be obtained from prospective studies in populations with clinical psychopathology. A Phase I/II study is ongoing with the inhaled GH001 formulation of 5-MeO-DMT in patients with Treatment-Resistant Depression (GH001-TRD-102; NCT04698603). This study aims to investigate safety, tolerability, and anti-depressive therapeutic effects of treatment with 5-MeO-DMT.

In conclusion, single doses of 6, 12, and 18 mg of the inhaled GH001 formulation of 5-MeO-DMT were able to induce a peak experience in a minority of healthy participants. An individualized dose escalation regimen produced a peak experience in every participant. Individualized dose escalation of 5-MeO-DMT dosing may be preferable for clinical applications that aim to maximize the short-term psychoactive effects to elicit a strong therapeutic response.

## Data Availability

The datasets presented in this article are not readily available because data are proprietary to GH Research. The datasets presented in this article are not readily available to protect proprietary information. Requests to access the datasets should be directed to clinicaltrials@ghres.com.
