# Supplementary material for: A Phase 1, Dose-Ranging Study to Assess Safety and Psychoactive Effects of a Vaporized 5-Methoxy-N, N-Dimethyltryptamine Formulation (GH001) in Healthy Volunteers
Source: Front Pharmacol. 2021 Nov 25;12:760671. doi: 10.3389/fphar.2021.760671 (PMC8667866; doi:10.3389/fphar.2021.760671)
Supplement: Supplementary file 1 [file DataSheet1.docx]

**A phase 1, dose-ranging study to assess safety and psychoactive effects of a vaporized 5-methoxy-N,N-dimethyltryptamine formulation (GH001) in healthy volunteers.**

**Supplementary information**

Reckweg J^1^, Mason NL^1^, van Leeuwen C^1^, Toennes SW^2^, Terwey TH^3^, Ramaekers JG^1^

^1^ Faculty of Psychology and Neuroscience, Maastricht University, Maastricht, The Netherlands

^2^ Institute of Legal Medicine, University of Frankfurt, Frankfurt/Main, Germany

^3^ GH Research, Dublin, Ireland

Correspondence: [j.ramaekers@maastrichtuniversity.nl](mailto:j.ramaekers@maastrichtuniversity.nl)

**Methods**

**Participants**

The participants needed to be free from psychotropic medication and had to have a body mass index between 18.5 and 27 kg/m². They needed to be healthy as determined by the medical supervisor, evaluated through vital signs, electrocardiogram (ECG), and laboratory parameters (hematology, clinical chemistry, and urinalysis) and show negative drug and pregnancy tests on the screening visit as well as administration day. A proficient understanding of English was required. Exclusion criteria included pregnancy, current use of dietary supplements (with the exception of vitamins) or psychoactive medication, a history of psychiatric disorders, current or previous drug abuse, a family history of a suspected or diagnosed genetic monoamine oxidase deficiency, and allergies or hypersensitivities or any other contraindication to 5-MeO-DMT.

**Procedure**

At visit A, the volunteers signed the informed consent and completed a medical screening, followed by a short practice session of the inhalation procedure. An intake interview structured around the Brief Psychiatric Rating Scale (BPRS) was completed by a psychologist and baseline assessments of subjective well-being and cognitive function were taken.

At visit B, participants were not allowed to have used alcohol on the day prior to the test day and on the test day and were not allowed to smoke during the test day. Participants had to stay fasted for a minimum of 4 hours before the start of the test day. After re-confirmation of participant eligibility, drug and alcohol screens were performed, as well as pregnancy tests for women. This was followed by setting up the Caretaker 4 system for vital parameter and ECG monitoring. About 30 minutes before administration, a baseline assessment of DASS-21, vital parameters and ECG were taken, and the participant was again informed about the procedure of the upcoming drug administration.

A pharmacokinetic blood sample was taken one hour and three hours after administration. Questionnaires about the psychedelic experience, subjective well-being, and the cognitive tests were performed from about 1.5 hours until about 3 hours after administration. At this time, blood and urine samples were taken. In addition, a structured discharge assessment was made by a psychologist who evaluated the state of mind of the participant based on a 30-minute closing out interview, structured around the BPRS. Subsequently, the medical supervisor determined whether the participant was fit to return home. The participants were requested to record any side effects until Visit D. For volunteers who participated in part B, this schedule, up to the discharge assessment, was repeated for each individual administration, omitting the one-hour post-administration blood sample.

The day after the administration day (Visit C), the participant was contacted via the phone to inquire if any side effects occurred after discharge and to inquire if any further assistance was required at this point. At the 1-week follow-up visit (Visit D, end-of-study), recording of side effects, another round of cognitive tests as well as the questionnaires about subjective well-being were performed. Lastly, another blood and urine sample for laboratory analyses were taken, before the participant was discharged.

**Subjective ratings of the psychedelic experience**

*Peak Experience Scale (PES)*

The primary assessment of the intensity of psychedelic effects relied on the proprietary PES, comprised of three questions (“How intense was the experience?”, “To what extent did you lose control?”, “How profound (i.e., deep and significant) was the experience?”), all answered by marking a Visual Analogue Scale between 0 and 100 mm, and then averaged to provide the total PE score. For the present analysis, a PE was pragmatically defined as the average score on the three questions greater than or equal to 75 mm.

*Duration of experience*

The duration of the experience (defined as time from drug administration to the time when any psychoactive symptoms had subsided), was independently recorded by the participant and the investigator.

*Ego Dissolution Inventory (EDI****)***

The EDI (Nour et al., 2016) is an 8-item self-report scale that assessed the participant’s experience of ego dissolution. Sample items for the scale included the following: “I experienced a dissolution of my “self” or ego” and “I felt at one with the universe”. The purpose of this scale was to acquire a better understanding of the experiences the participant had about ego dissolution during the psychedelic experience. The participant answered the scale with endpoints of either 0 = “No, not more than usually” or 100 = “Yes, I experience this completely/entirely”. The EDI is scored by calculating the mean of all the 8 items (range 0-100). The higher the total score, the stronger the experience of ego dissolution.

*Mystical Experiences Questionnaire (MEQ)*

The MEQ(Studerus et al., 2010;Barrett et al., 2015) contains 30 items from the previous 43-item version (MEQ-43). The four factors of the MEQ are: mystical (including items from the internal unity, external unity, noetic quality, and sacredness scales of the MEQ-43), positive mood, transcendence of time and space, and ineffability (all three of which include items from their respective MEQ-43 scales). Thus, the MEQ retained items from each qualitative subscale in the original MEQ-43, but in a reduced number of dimensions. Responses were indicated on a 6-point scale ranging from “None; not at all” to “Extreme”.

*Challenging Experiences Questionnaire (CEQ)*

The CEQ (Barrett et al., 2016) is comprised of 26 items (e.g., “Sadness”, “Feelings of Despair”, “I felt isolated from everything and everyone”) that make up seven subscales (grief, fear, death, insanity, isolation, physical distress, and paranoia) to provide a degree to which a given psychedelic experience was challenging for the participant. The participant indicated the appropriate response on a 6-point scale, ranging from “None; not at all” to “Extreme”. Response data were divided by the maximum response (i.e., 5). The average of all transformed item scores across all items was computed to obtain the CEQ total score.

*5-Dimensional Altered States of Consciousness Questionnaire (5D-ASC)*

The 5D-ASC (Dittrich, 1998;Studerus et al., 2010) is a 94-item self-report scale that assessed the participant’s alterations from normal waking consciousness. The participant was asked to make a vertical mark on a line below each statement in order to rate to what extent the statements applied to their experience in retrospect (e.g., from “No, not more than usually” to “Yes, more than usually”). The 5D-ASC measures 11 subscales: experience of unity, spiritual experience, blissful state, insightfulness, disembodiment, impaired control and cognition, anxiety, complex imagery, elementary imagery, audio-visual synesthesia and changed meaning of percepts. Alternatively, the scores can also be summarized in 5 key-dimensions: Oceanic Boundlessness, Anxious Ego Dissolution, Visual Restructuralization, Auditory Alterations, and Reduction of Vigilance.

**Subjective measures of mood and well-being**

*Depression, Anxiety and Stress Scale (DASS-21)*

The DASS-21 (Henry and Crawford, 2005) is a shorter version of the originally 42-item long self-report questionnaire. Sample items for each subscale included the following: “I couldn’t seem to experience any positive feeling at all”, “I was aware of dryness of my mouth”, and “I found it hard to wind down”, respectively. The purpose of this scale was to measure constructs of depression, anxiety, and stress. The participants responded by rating the concordance with each statement from 0 (did not apply to me at all) to 3 (applied to me very much, or most of the time). The subscale scores for depression, anxiety and stress were calculated by summing the scores for the relevant items, multiplied by 2, given that the original DASS has 42 questions.

*Satisfaction With Life Scale (SWLS****)***

The SWLS (Diener et al., 1985) is a 5-item self-report scale. Sample items included: “In most ways my life is close to my ideal” and “So far I have gotten the important things I want in life “. The purpose of the scale was to assess someone’s satisfaction with life. The items were answered on a Likert scale ranging from 1, “Strongly disagree” to 7, “Strongly agree”. The total score was obtained by summarizing the ratings from each item and ranges between 5 to 35, with higher scores indicating a greater life satisfaction.

*Five Facets Mindfulness Questionnaire (FFMQ)*

The FFMQ (Baer et al., 2006) is a 39-item self-report questionnaire assessing five different factors 1) Observe: noticing experience that are both internal and external such as for example thoughts and emotions, e.g., “When I’m walking, I deliberately notice the sensations of my body moving”; 2) Describe: describing internal experiences, e.g., “I’m good at finding words to describe my feelings”; 3) Acting with awareness: focus on the present activity, e.g., “When I do things, my mind wanders off and I’m easily distracted”; 4) Non-judgment: not evaluating the present experience, e.g., “I criticize myself for having irrational or inappropriate emotions”; 5) Non-reaction: allowing thoughts and feelings to come without acting or reacting upon them, e.g.,“ I perceive my feelings and emotions without having to react to them”. The purpose of this scale was to obtain an understanding of an individual’s mindfulness-related capacities. The participants answered the FFMQ by rating the concordance with each statement on a 5-point Likert-scale that ranged from 1 (never true) to 5 (very often or always true). The total FFMQ score is obtained by adding the subscale scores.

*Clinician Administered Dissociative States Scale (CADSS)*

The CADSS (Bremner et al., 1998) comprised 19 subjective items, ranging from 0 ‘not at all’ to 4 ‘extremely’. It is divided into 3 components: 1) depersonalization, 2) derealization and 3) amnesia. Summed together, these subscales formed a total dissociative score. The CADSS was specifically designed to be a standardized measure of present-state dissociative symptomatology.

*Brief Psychiatric Rating Scale* *(BPRS)*

The BPRS (Overall and Gorham, 1962) was intended to screen for psychiatric symptoms in a structured fashion and was evaluated at the screening visit and before the participant left the site on the study drug administration day (as part of the ‘closing-out interview’). Each symptom was rated 1-7 and a total of 18 symptoms were scored.

**Cognitive tests**

*Psychomotor Vigilance Task (PVT)*

The PVT (Lim and Dinges, 2008) assessed the reaction time (RT) in response to a visual stimulus. The visual stimulus was a counter in the middle of a computer screen that started at random intervals between 2 and 10 seconds. The participant responded by pressing a button as soon as possible after the counter started. Duration of the task was 6 minutes. The process was used to measure sustained attention performance. Outcome measure was the number of attentional lapses (RT ≥ 500 msec).

*The Prospective Memory Task (PMT)*

The PMT (Ramaekers et al., 2009) examines prospective memory performance in an event-based memory task. The foreground task consisted of 240 successive presentations of a letter (A or B) in the center of a computer screen. Subjects were required to respond to each letter as quickly as possible by pressing one of two response buttons. One button was pressed to indicate that the letter ‘A’ appeared and the other to indicate the letter ‘B’. Both letters were presented equally often. Participants were informed about the trial number by means of a trial counter that was always present in the top left corner of the screen. In addition, participants were presented at irregular times with a future trial number in the right top corner of the display. Participants were instructed to remember this future trial number and withhold from responding to the foreground task during the actual occurrence of the future trial. The memory set of trial numbers was dynamic and contained up to three future trial numbers. A novel future trial number replaced a trial number in the memory set, whenever the actual trial number matched a future trial number in the set. Trials during which participants were expected to respond were classified as Go trials. Trials during which participants were instructed to withhold a response were classified as No-Go trials (prospective memory trials). Time between presentation of a future trial number and the actual occurrence of the trial (i.e., memory delays) varied between 30, 60 and 90 s, and was equally divided over all No-Go trials. In total, the prospective memory task consisted of 216 Go trials and 24 No-Go trials. The dependent variable was the number of Correct Prospective Memory Recalls (i.e., number of correct response inhibitions) in the No-Go trials.

*Digit symbol substitution task (DSST)*

The DSST (Royer and Janowitch, 1973) was a computerized version of the original paper and pencil test taken from the Wechsler Adult Intelligence Scale. The participant was shown an encoding scheme consisting of a row of squares at the top of the screen, wherein nine digits were randomly associated with particular symbols. The same symbols were presented in a fixed sequence at the bottom of the screen as a row of separate response buttons. The randomization procedure was chosen such that symbols never appeared at the same ordinal position within both rows. The encoding scheme and the response buttons remained visible while the participant was shown successive presentations of a single digit at the center of the screen. The task was to match each digit with a symbol from the encoding list and click the corresponding response button. The percentage of digits correctly encoded among the total number of responses within 3 minutes was the performance measure.

Table S1 Participants’ experience with previous drug use.

| **Substance** | **Total (*N*=22)** | **Dose** | | | | | | | | |
| --- | --- | --- | --- | --- | --- | --- | --- | --- | --- | --- |
|  |  | **2 mg (*n*=4)** | | **6 mg (*n*=6)** | | **12 mg (*n*=4)** | | **18 mg (*n*=4)** | | **IDE**  **(*n*=4)** |
| Alcohol | 19 | | 3 | | 6 | | 3 | | 3 | 4 |
| Cannabis | 18 | | 2 | | 6 | | 2 | | 4 | 4 |
| Psilocybin | 21 | | 4 | | 5 | | 4 | | 4 | 4 |
| LSD | 16 | | 2 | | 4 | | 3 | | 3 | 4 |
| DMT | 5 | | 1 | | 0 | | 0 | | 2 | 2 |
| 5-MeO-DMT | 4 | | 0 | | 0 | | 2 | | 1 | 1 |
| Ayahuasca | 8 | | 1 | | 0 | | 3 | | 1 | 3 |
| Salvia | 2 | | 1 | | 0 | | 0 | | 1 | 0 |
| 2-CB) | 2 | | 0 | | 0 | | 2 | | 0 | 0 |
| Mescaline | 1 | | 0 | | 0 | | 0 | | 1 | 0 |
| Changa | 1 | | 0 | | 0 | | 0 | | 0 | 1 |
| DOC) | 1 | | 0 | | 0 | | 0 | | 0 | 1 |
| MDMA | 17 | | 3 | | 5 | | 3 | | 3 | 3 |
| Amphetamine | 7 | | 1 | | 0 | | 2 | | 2 | 2 |
| Cocaine | 9 | | 1 | | 3 | | 2 | | 1 | 2 |
| Ketamine | 5 | | 0 | | 0 | | 2 | | 3 | 0 |
| Opioids (unspecified) | 1 | | 0 | | 0 | | 0 | | 1 | 0 |

Table S2 ANOVA contrast of psychedelic ratings during incremental dose conditions relative to the lowest dose of 5-MeO-DMT. P-values in Bold survive sequential Bonferroni corrections for multiple comparisons. (^a^ bounded maximum)

| **Ratings of the psychedelic experience** | **Contrast levels** | **Difference** | **SE** | ***p*** | **95% confidence intervals** | |
| --- | --- | --- | --- | --- | --- | --- |
|  |  |  |  |  | **Lower bound** | **Upper bound** |
| PES | 6mg vs 2mg | 34.779 | 12.657 | **0.014*** | 8.074 | 61.483 |
|  | 12mg vs 2mg | 56.501 | 13.865 | **0.001*** | 27.248 | 85.754 |
|  | 18mg vs 2mg | 50.168 | 13.865 | **0.002*** | 20.914 | 79.421 |
|  | IDE vs 2mg | 80.335 | 13.865 | **< 0.001**** | 51.082 | 100.00^a^ |
| EDI | 6mg vs 2mg | 16.260 | 12.284 | 0.203 | -9.656 | 42.177 |
|  | 12mg vs 2mg | 20.626 | 13.456 | 0.144 | -7.763 | 49.016 |
|  | 18mg vs 2mg | 25.781 | 13.456 | 0.072 | -2.608 | 54.171 |
|  | IDE vs 2mg | 66.813 | 13.456 | **< 0.001**** | 38.423 | 95.202 |
| MEQ (total average) | 6mg vs 2mg | 1.244 | 0.510 | 0.026* | 0.169 | 2.320 |
|  | 12mg vs 2mg | 1.783 | 0.558 | **0.005*** | 0.605 | 2.962 |
|  | 18mg vs 2mg | 1.266 | 0.558 | **0.037*** | 0.088 | 2.444 |
|  | IDE vs 2mg | 3.092 | 0.558 | **< 0.001**** | 1.913 | 4.270 |
| CEQ (total average) | 6mg vs 2mg | 0.071 | 0.060 | 0.253 | -0.056 | 0.198 |
|  | 12mg vs 2mg | 0.020 | 0.066 | 0.760 | -0.119 | 0.160 |
|  | 18mg vs 2mg | 0.047 | 0.066 | 0.486 | -0.092 | 0.186 |
|  | IDE vs 2mg | 0.044 | 0.066 | 0.513 | -0.095 | 0.183 |
| 5D-ASC % Oceanic Boundlessness | 6mg vs 2mg | 19.05 | 11.54 | 0.117 | -5.29 | 43.39 |
|  | 12mg vs 2mg | 15.50 | 12.64 | 0.237 | -11.16 | 42.17 |
|  | 18mg vs 2mg | 23.76 | 12.64 | 0.077 | -2.90 | 50.43 |
|  | IDE vs 2mg | 42.16 | 12.64 | **0.004*** | 14.49 | 68.24 |
| 5D-ASC % Anxious Ego Dissolution | 6mg vs 2mg | 11.42 | 7.56 | 0.149 | -4.53 | 27.37 |
|  | 12mg vs 2mg | 13.02 | 8.28 | 0.134 | -4.45 | 30.49 |
|  | 18mg vs 2mg | 12.77 | 8.28 | 0.141 | -4.7 | 30.25 |
|  | IDE vs 2mg | 16.905 | 8.28 | 0.057 | -0.57 | 34.38 |
| 5D-ASC % Visual Restructuralization | 6mg vs 2mg | 15.93 | 6.55 | 0.026* | 2.12 | 29.74 |
|  | 12mg vs 2mg | 4.05 | 7.17 | 0.579 | -11.08 | 19.18 |
|  | 18mg vs 2mg | 9.33 | 7.17 | 0.211 | -5.8 | 24.46 |
|  | IDE vs 2mg | 16.873 | 7.17 | 0.031* | 1.74 | 32.00 |
| 5D-ASC % Auditory Alterations | 6mg vs 2mg | 6.95 | 4.17 | 0.114 | -1.85 | 15.76 |
|  | 12mg vs 2mg | 0.72 | 4.57 | 0.877 | -8.92 | 10.36 |
|  | 18mg vs 2mg | 1.34 | 4.57 | 0.772 | -8.3 | 10.99 |
|  | IDE vs 2mg | 4.97 | 4.57 | 0.292 | -4.67 | 14.61 |
| 5D-ASC % Reduction of Vigilance | 6mg vs 2mg | 26.35 | 7.56 | **0.003*** | 10.41 | 42.29 |
|  | 12mg vs 2mg | 9.94 | 8.28 | 0.246 | -7.52 | 27.4 |
|  | 18mg vs 2mg | 25.793 | 8.28 | **0.006*** | 8.33 | 43.25 |
|  | IDE vs 2mg | 18.96 | 8.28 | 0.035* | 1.5 | 36.42 |

**References**

Baer, R.A., Smith, G.T., Hopkins, J., Krietemeyer, J., and Toney, L. (2006). Using self-report assessment methods to explore facets of mindfulness. *Assessment* 13**,** 27-45.

Barrett, F.S., Bradstreet, M.P., Leoutsakos, J.-M.S., Johnson, M.W., and Griffiths, R.R. (2016). The Challenging Experience Questionnaire: Characterization of challenging experiences with psilocybin mushrooms. *Journal of Psychopharmacology* 30**,** 1279-1295.

Barrett, F.S., Johnson, M.W., and Griffiths, R.R. (2015). Validation of the revised Mystical Experience Questionnaire in experimental sessions with psilocybin. *Journal of Psychopharmacology* 29**,** 1182-1190.

Bremner, J.D., Krystal, J.H., Putnam, F.W., Southwick, S.M., Marmar, C., Charney, D.S., and Mazure, C.M. (1998). Measurement of dissociative states with the clinician‐administered dissociative states scale (CADSS). *Journal of Traumatic Stress: Official Publication of The International Society for Traumatic Stress Studies* 11**,** 125-136.

Diener, E., Emmons, R.A., Larsen, R.J., and Griffin, S. (1985). The satisfaction with life scale. *Journal of personality assessment* 49**,** 71-75.

Dittrich, A. (1998). The standardized psychometric assessment of altered states of consciousness (ASCs) in humans. *Pharmacopsychiatry* 31 Suppl 2**,** 80-84.

Henry, J.D., and Crawford, J.R. (2005). The short‐form version of the Depression Anxiety Stress Scales (DASS‐21): Construct validity and normative data in a large non‐clinical sample. *British journal of clinical psychology* 44**,** 227-239.

Lim, J., and Dinges, D.F. (2008). Sleep deprivation and vigilant attention. *Annals of the New York Academy of Sciences* 1129**,** 305-322.

Nour, M.M., Evans, L., Nutt, D., and Carhart-Harris, R.L. (2016). Ego-dissolution and psychedelics: validation of the ego-dissolution inventory (EDI). *Frontiers in human neuroscience* 10**,** 269.

Overall, J.E., and Gorham, D.R. (1962). The brief psychiatric rating scale. *Psychological reports* 10**,** 799-812.

Ramaekers, J.G., Kuypers, K.P., Wingen, M., Heinecke, A., and Formisano, E. (2009). Involvement of inferior parietal lobules in prospective memory impairment during acute MDMA (ecstasy) intoxication: an event-related fMRI study. *Neuropsychopharmacology* 34**,** 1641-1648.

Royer, F.L., and Janowitch, L. (1973). Performance of process and reactive schizophrenics on a symbol-digit substitution task. *Perceptual and Motor Skills* 37**,** 63-70.

Studerus, E., Gamma, A., and Vollenweider, F.X. (2010). Psychometric evaluation of the altered states of consciousness rating scale (OAV). *PloS one* 5**,** e12412.
